# Corneal Curvature of the Saudi Population in Relation to Age, Gender, Refractive Errors, and Central Corneal Thickness

**DOI:** 10.7759/cureus.73845

**Published:** 2024-11-17

**Authors:** Abdulrahman A Almazrou, Amani Abualnaja, Wala Abualnaja, Nadeef Alqahtani, Ahmed Z Alkhars

**Affiliations:** 1 Ophthalmology, Imam Mohammad Ibn Saud Islamic University, Riyadh, SAU; 2 Internal Medicine, King Faisal Specialist Hospital & Research Centre, Riyadh, SAU; 3 Optometry, King Fahd District Health Center, Riyadh, SAU; 4 Physical Medicine and Rehabilitation, Imam Mohammad Ibn Saud Islamic University, Riyadh, SAU; 5 General Physician, AlJaber Eye and ENT Hospital, Al-Ahsa, SAU

**Keywords:** central corneal thickness, cornea, corneal curvature, refractive errors, saudi

## Abstract

Corneal curvature (CC) is a crucial ocular optical system parameter, indicating the shape of the cornea expressed in radii. This study aims to investigate the relationship between CC, age, gender, refractive errors, and central corneal thickness (CCT) in Saudi Arabia, a field lacking in research. Data were gathered from Imam Medical Center in Riyadh and Dr. Sulaiman Al Habib Hospital in this randomized, hospital-based, retrospective analysis. Patients sent to the refractive surgery clinic for a laser-assisted in situ keratomileusis (LASIK) evaluation provided a total of 1,005 eyes for the study. Patients with stable refractions in all groups and those between the ages of 17 and 57 with no history of systemic illness, eye operations, or ocular pathology were included in the research, as were all groups with stable refractions. An ultrasound pachymeter was used to assess the CC and CCT. This study showed a mean CC of 42.93+/-1.4 at a mean age of 28.93+/-7.09. We found a significant negative, weak correlation between mean CC and CCT. Gender was not significantly associated with CC in myopic and hyperopic patients, while in patients with astigmatism, females were observed to have a significantly higher mean CC compared to males. A significant difference in the mean CC was observed across different refractive errors, and patients with astigmatism had the highest CC, followed by myopic and hyperopic patients.

## Introduction

The cornea is an avascular clear transparent part of the eye that covers the iris and the pupil, focusing light inside the eye [1]. The cornea is composed of six layers: epithelium, Bowman's layer, stroma, Dua's layer, Descemet's membrane, and endothelium [1]. The importance of the cornea is evident, as the diseases affecting the cornea are a major cause of blindness worldwide [2]. The epidemiology of corneal blindness is complicated and includes a wide assortment of infectious and inflammatory eye diseases that cause cornea scarring, leading to functional blindness [2]. The anterior corneal surface is an important component of the eye's dioptric power, accounting for 80% of total refractive power [3].

Also, the corneal surface can have a major impact on vision acuity and quality [4]. Research shows that any changes in the sphericity of the corneal surface can lead to drastic shifts in the eye's refractive power [4-5].

Corneal parameters such as corneal diameter, corneal curvature (CC), and central corneal thickness (CCT) are essential for both diagnostic and therapeutic purposes [6]. Corneal curvature is one of the most important parameters in the ocular optical system, and it measures the shape of the cornea expressed in radii [6]. Any disparity in corneal curvature between the corneal meridians is the cause of corneal astigmatism [6]. Corneal curvature is important in detecting many eye conditions like keratoconus, keratoglobus, and Marfan's syndrome [6]. This important parameter is used for IOL calculation in cataract surgery and refractive surgery and is helpful in contact lens fitting [6].

The study also revealed that the corneal surface is affected more in females than in males in the specified age group; this information is important to include to represent the full result of this study.

The cohort study conducted by Hashemi et al. in Iran between 2009 and 2014, aiming to determine age-related changes in corneal curvature and shape, showed that between 40 and 64-year-olds, corneal changes are in line with maintaining a prolate ellipsoid shape of the cornea [7]. It also showed that the anterior surface changes more than the posterior and that the corneal surface is more affected in females than males [7].

In 2001, a study done by Goto T et al. aimed to investigate gender- and age-related differences in the corneal topography of a normal population showed that the corneas of older men were flatter than those of older women (p < 0.001, and showed statistically significant gender-related changes with aging (p < 0.001) [8].

Another study by AlMahmoud T et al. showed that in the myopic group, an inverse relationship between spherical equivalent and mean keratometry was seen. In hyperopes, a significant correlation between the cycloplegic spherical equivalent and keratometry was also seen [9].

The Chen et al. study found no significant relationship between mean corneal curvature and central corneal thickness in normal Taiwanese Chinese people aged 40-80 (Pearson r=0.013; P=0.770) [10]. Due to the lack of studies in this field in Saudi Arabia, this study aims to investigate the relationship between Corneal curvature and age, gender, refractive errors, and central corneal thickness.

## Materials and methods

Study design

The purpose of this hospital-based, retrospective, randomizing study was to investigate the relationship between corneal curvature and age, gender, refractive error, and central corneal thickness. The study was conducted at Imam Medical Center, and Dr. Sulaiman Al-Habib, Takhassusi Street, Riyadh, Saudi Arabia, and the data was collected from the period March 2022 to December 2022. The study was conducted in adherence to the principles of the 2013 Declaration of Helsinki.

Participants and procedures

This study included patients referred to the refractive surgery clinic for LASIK assessment or who had undergone refractive surgery. Patients between the ages of 17 and 57 with no history of ocular disease, no systemic disease, and refractive stability were included. However, this study excluded patients aged less than 17 years and patients with corneal pathology such as edema, scarring, or corneal dystrophy, a history of refractive or ocular surgery, a history of ocular trauma, and glaucomatous eyes. Patients with systemic diseases like diabetes or rheumatoid arthritis and pregnant were also excluded. The data was collected from Dr. Suliman Al Habib Specialist Hospital and Imam Medical Center, Riyadh, Saudi Arabia. Sample size calculations were performed using the following formula: n = z^2^pq\d^2^. The confidence level was 95%, estimated proportions of 50% were used, the precision level was 5%, the estimated population was 5368, and the minimum required sample size was calculated to be 359. The population estimation was done as follows: first, the center had an average of 8 surgeries per week, taking into account that both eyes were involved, the average number of surgeries per year was 864. In the second center, 42 surgeries were performed weekly, making the average number of surgeries yearly 4536. Summed together, an estimated population of 5368 from both centers was calculated. The identifying data were age and gender, while the examination data were measured in the study were CCT, refraction, and corneal curvature. CCT was measured with an ultrasonic pachymeter, refracted using an automatic refractor, and subjectively confirmed by audition and retinoscopy. Refraction in D was calculated as spherical equivalent (SE), which is ((Spherical Equivalence (SE) = sphere + Cylinder/2)). Corneal curvature was measured using an automatic refractive keratometer, which measured two major corneal radii (k1 and k2) 90° apart, and the average of the two values will be recorded as the mean corneal curvature (AVK).

Statistical analysis

Data analysis was performed using Statistical Package for the Social Sciences, SPSS 23rd version. Frequency and percentages were used to display categorical variables. Mean and standard deviation were used to display numerical variables. The chi-square test was used to test for the presence of an association between categorical variables. Independent t-test and ANOVA test were also used to test for factors associated with mean corneal curvature. ANOVA test followed the Tukey post-hoc test to determine where the exact differences between subgroups exist. Pearson's correlation was also used to test for correlation between numerical variables. The level of significance was set at 0.05

## Results

A total of 946 eyes were included in the study. Table 1 displays the overall patient's profile. As for the gender, the eyes of 363 (38.4%) of the patients belonged to males, and the eyes of 583 (61.6%) belonged to females. 466 (49.3%) of the included eyes were left eyes, and 480 (50.7%) of the included eyes were right eyes. As for the center from which the participants were recruited, 829 (87.6%) were recruited from Dr. Suliman Al Habib Specialist Hospital, and 117 (12.4%) were recruited from Imam Medical Center. The mean age of the patients was 28.93+/-7.09. The mean of central corneal thickness was 547.92+/-30.48.

**Table 1:** Overall patients profile (n = 946)

Categorical Characteristics	n	%
Gender		
Male	363	38.40
Female	583	61.60
Eye		
OS	466	49.30
OD	480	50.70
Hospital		
Dr. Sulaiman Al-Habib	829	87.60
Imam Medical Center	117	12.40
Numerical Characteristics	Mean	Standard deviation
Age	28.93	7.09
Central corneal thickness	547.92	30.48

**Figure 1:**
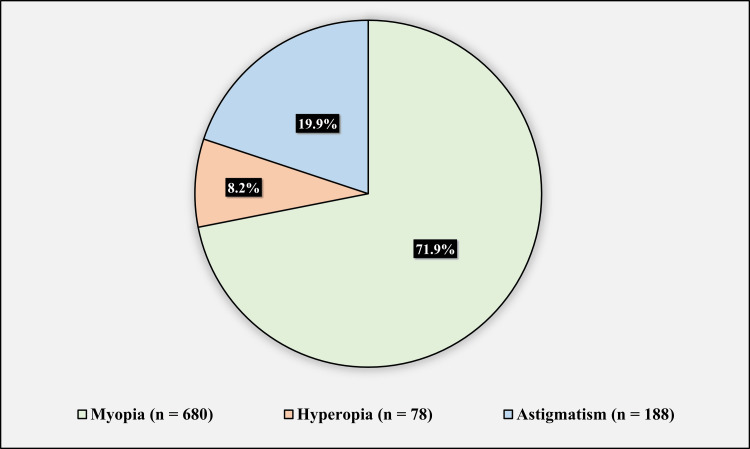
Participants’ refractive errors

Figure 1 shows the participants’ refractive error. Six hundred eighty (71.9%) of patients had myopia, 78 (8.2%) of patients had hyperopia, and 188 (19.9%) of patients had astigmatism

**Figure 2:**
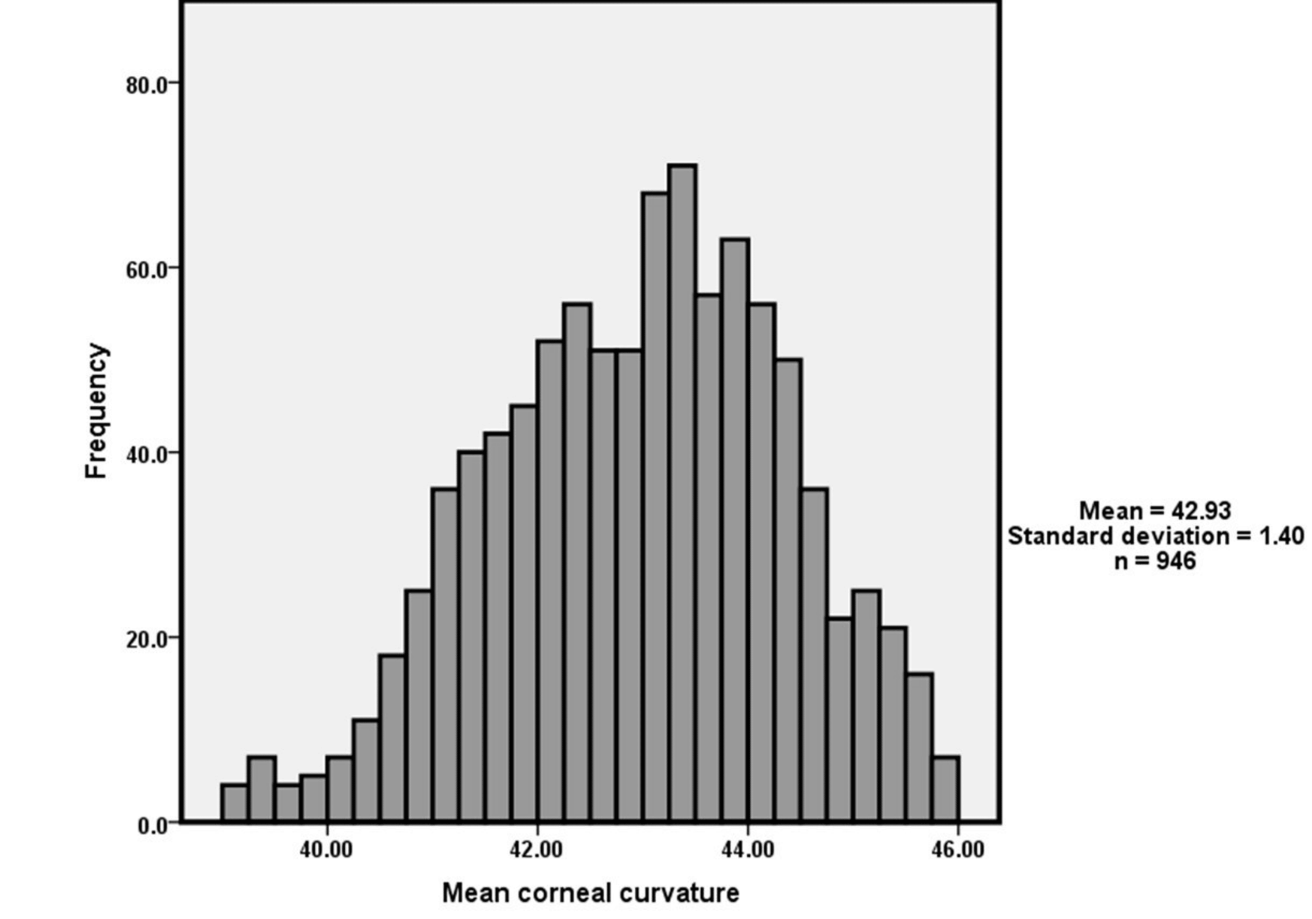
Distribution of mean corneal curvature measurement

Figure 2 demonstrates the distribution of mean corneal curvature. The participants mean of mean corneal curvature was 42.93+/-1.4.

Table 2 demonstrates the factors associated with mean corneal curvature within each type of refractive error respectively. As for the association testing in patients with myopia, a significant difference was observed in mean corneal curvature was observed across genders (p < 0.001), where it was observed that females had a significantly higher mean corneal curvature compared to males (43.15+/-1.4 vs 42.63+/-1.37). Moreover, a significant negative, weak correlation was found between mean corneal curvature and central corneal thickness (p = 0.023, Pearson's correlation = - 0.087). In myopic patients, age, side, and sphere equivalent were not significantly associated with mean corneal curvature. As for the association testing in patients with hyperopia, a significant negative positive correlation was found between mean corneal curvature and age (p = 0.031, Pearson's correlation = 0.245). In hyperopic patients, gender, side, sphere equivalent, and central corneal thickness were all not significantly associated with mean corneal curvature. As for the association testing in patients with astigmatism, gender was significantly associated with mean corneal curvature (p = 0.009), whereas females were observed to have significantly higher mean corneal curvature compared to males (43.29+/-1.39 vs 42.8+/-1.19). In patients with astigmatism, age, side, central corneal thickness, sphere power, mean, astigmatism degree, and axis were all not significantly associated with mean corneal curvature.

**Table 2:** Factors associated with mean corneal curvature within each type of refractive error, respectively *Significance level 0.05

Factor		Mean Corneal Curvature		P-Value
		Mean	Standard Deviation	
Association Testing in Patients with Myopia (Part 1)				
Gender				< 0.001*
Male		42.63	1.37	
Female		43.15	1.40	
Eye				0.678
OS		42.94	1.40	
OD		42.99	1.43	
Association Testing in Patients with Hyperopia (1)				
Gender				0.218
Male		42.12	1.44	
Female		42.52	1.32	
Eye				0.880
OS		42.40	1.36	
OD		42.36	1.39	
Association Testing in Patients with Astigmatism (Part 1)				
Gender				0.009*
Male		42.8	1.19	
Female		43.29	1.39	
Eye				0.533
OS		43.10	1.29	
OD		42.99	1.35	
Association Testing in Patients with Myopia (Part 2)				
Age correlation with mean corneal curvature				
P-value			0.273	
Pearson's Correlation			-0.420	
Central corneal thickness correlation with mean corneal curvature				
P-value			0.023*	
Pearson's Correlation			-0.087	
Sphere equivalent correlation with mean corneal curvature				
P-value			0.128	
Pearson's Correlation			-0.058	
Association Testing in Patients with Hyperopia ((Part 2)				
Age correlation with mean corneal curvature				
P-value			0.031*	
Pearson's Correlation			0.245	
Central corneal thickness correlation with mean corneal curvature				
P-value			0.722	
Pearson's Correlation			-0.041	
Sphere equivalent correlation with mean corneal curvature				
P-value			0.153	
Pearson's Correlation			-0.163	
Association Testing in Patients with Astigmatism (Part 2)				
Age correlation with mean corneal curvature				
P-value			0.186	
Pearson's Correlation			0.097	
Central corneal thickness correlation with mean corneal curvature				
P-value			0.480	
Pearson's Correlation			-0.052	
Sphere Power correlation with mean corneal curvature				
P-value			0.528	
Pearson's Correlation			-0.052	
Astigmatism correlation with mean corneal curvature				
P-value			0.135	
Pearson's Correlation			-0.109	
Axis correlation with mean corneal curvature				
P-value			0.690	
Pearson's Correlation			-0.029	

Table 3 illustrates the comparison of mean corneal curvature across refractive error type. A significant difference in the mean of mean corneal curvature was observed across different refractive errors (p = 0.001). The highest mean corneal curvature was observed in patients with astigmatism (43.04 +/- 1.32), followed by patients with myopia (42.97 +/- 1.41), while patients with hyperopia had the lowest mean corneal curvature (42.38). Tukey post-hoc test revealed that patients with hyperopia had significantly lower mean corneal curvature compared to those with myopia and compared to those with astigmatism (p < 0.05).

**Table 3:** Comparison of mean corneal curvature across refractive error type *Significance level 0.05

Factor	Central Corneal Curvature		P-Value
	Mean	Standard Deviation	
Gender			0.001*
Myopia	42.97	1.41	
Hyperopia	42.38	1.36	
Astigmatism	43.04	1.32	

## Discussion

The findings of this retrospective study evaluated the corneal curvature in a typical Saudi population and examined the relationship between corneal curvature and age, gender, and refraction. The mean corneal curvature was 42.93+/-1.4 at the mean age of (28.93+/-7.09).

Our study showed a significant negative, weak correlation between mean corneal curvature and central corneal thickness (p = 0.023, Pearson's correlation = - 0.087). Khadim et al. have also found a statistically significant (weak) negative correlation (Pearson r=−0.097, P=0.048) between CCT and corneal curvature in their study done in Iraq on healthy individuals aged from 20 to 75 [11]. However, according to Chen et al., CCT was not associated with refractive error, corneal curvature, anterior chamber depth, and axial length. CCT was found to be an independent factor unrelated to other parameters [10].

Another difference was observed in mean corneal curvature across gender (p < 0.001), where it was observed that females had a significantly higher mean corneal curvature compared to males, which was found to be (43.15+/-1.4) and (42.63+/-1.37) respectively. A similar result was established by Iyamu et al.; their study found that females had slightly higher mean average corneal curvature (AVK) than males. However, age, age group, and gender did not significantly affect corneal curvature [12].

Regarding the relation between corneal curvature and age, in contrast to our findings, a study titled "Age-Related Changes in Corneal Curvature and Shape: The Shahroud Eye Cohort Study" revealed that there was an age-related decrease in the corneal radius of curvature from the center up to the 5 to 7-mm ring and an increased corneal radius of curvature [7].

In relation to refractive errors, a significant difference in the mean of mean corneal curvature was observed across different refractive errors (p = 0.001). The highest mean corneal curvature was observed in patients with astigmatism (43.04+/-1.32), in which gender was significantly associated with mean corneal curvature (p = 0.009), females were observed to have significantly higher mean corneal curvature compared to males (43.29+/-1.39 vs 42.8+/-1.19). Age, side, central corneal thickness, sphere power, mean, astigmatism degree, and axis were all not significantly associated with mean corneal curvature.

In myopic patients, age, side, and sphere equivalent were not significantly associated with mean corneal curvature, while patients with hyperopia had the lowest mean corneal curvature (42.38). Tukey post-hoc test revealed that patients with hyperopia had significantly lower mean corneal curvature compared to those with myopia and compared to those with astigmatism (p < 0.05). In hyperopic patients, gender, side, sphere equivalent, and central corneal thickness were all not significantly associated with mean corneal curvature. This result is in accordance with the observations made in similar studies [7,12-13].

Limitations and recommendations

The intra-ocular pressure was not obtained. However, a masked prospective clinical trial done by Kohlhaas et al. demonstrated no significant association between intraocular pressure readings and corneal curvature or axial length [14]. We recommend performing a nationwide study that includes participants with different sociodemographic and medical characteristics to establish a holistic view of the country's general population corneal parameters status.

## Conclusions

This study showed a mean corneal curvature of 42.93+/-1.4 at the mean age of (28.93+/-7.09). We found a significant negative, weak correlation between mean corneal curvature and central corneal thickness. Gender was not significantly associated with corneal curvature in myopic and hyperopic patients, while in patients with astigmatism, females were observed to have a significantly higher mean corneal curvature compared to males. In addition, there was no significant association between mean corneal curvature and CCT in all three groups. A significant difference in the mean corneal curvature was observed across different refractive errors, and patients with astigmatism had the highest corneal curvature, followed by myopic and hyperopic patients.
